# Feeder Insects: The Ethics of Live Feeding in Zoos and Aquariums

**DOI:** 10.1002/zoo.21902

**Published:** 2025-04-22

**Authors:** Bob Fischer

**Affiliations:** ^1^ Texas State University San Marcos Texas USA

**Keywords:** 3Rs, animal welfare, ethics, insects, precautionary reasoning

## Abstract

While there is no consensus about whether insects are sentient, the possibility alone raises an important question for zoos and aquariums. What ethical responsibilities, if any, do zoos and aquariums have concerning their feeder insects? There has been very little scholarly discussion of these questions. This is not surprising, as scholars have largely ignored feeder animals. So, this paper takes up two tasks. First, it surveys the main welfare‐focused ethical questions associated with feeder animals generally and feeder insects in particular. The aim here is to identify the main considerations that bear on a full assessment of the ethics of using feeder animals as a resource, thereby facilitating future research. Second, in the interest of making practical recommendations in the face of significant moral complexity and uncertainty, this paper adopts a standard institutional ethical framework—the 3Rs—and considers its implications for the use of feeder insects.

## Graphical abstract

Since there is uncertainty about whether some feeder insects are sentient, zoos and aquariums should consider their welfare alongside the welfare of the animals to which they are fed. The 3Rs (replacement, reduction, refinement) provide a useful ethical framework for identifying best practices.

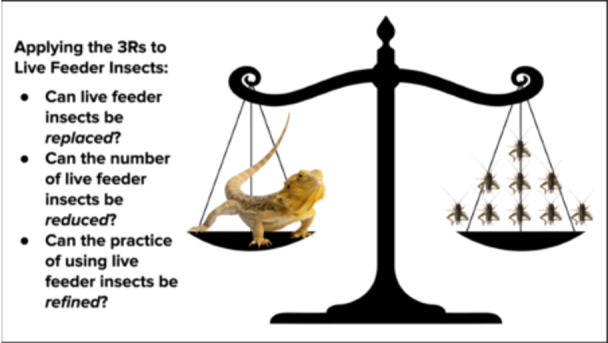

## 1 Introduction

Zoos keep many obligate carnivores. Some of those animals will not feed unless they have the opportunity to catch and kill live prey. Many others prefer live prey, such that providing it is considered an enrichment. So, despite some risks associated with live feeding—most obviously, the defensive efforts of the prey animals—it is a common practice at many institutions.

Many of these “feeder” animals—that is, the prey animals supplied to predators—are insects. According to the Smithsonian's National Zoo & Conservation Biology Institute, for instance,

A mind‐boggling number of bugs are consumed by the Zoo's insectivores, or insect‐eating animals, each week: 121,000 crickets, 150,000 mealworms, and 17,000 waxworms, not including thousands of other non‐insect invertebrates, like earthworms and springtails. Put differently, the Zoo's animals go through about 80 pounds (36 kilograms) of crickets and about 30 pounds (13 kilograms) of mealworms each week. Combined, that's roughly the weight of a small human adult (SNZCBI 2024).

These insects (and other invertebrates, though we'll bracket those others here) are not covered by the American Zoological Association's guidelines for the humane management of feeder animals, which are limited to terrestrial vertebrates (AZA Nutrition Scientific Advisory Group 2017). The omission is understandable given doubts about whether insects are *sentient*—that is, whether they have the capacity to feel pain and have other valenced experiences. However, the scientific landscape has changed rapidly in recent years, with an increasing number of researchers suggesting that insect sentience is a realistic possibility that deserves serious consideration (Barron and Klein 2016; Fischer and Larson 2019; Mikhalevich and Powell 2020; Gibbons et al. 2022; Barrett 2024; Birch 2024).

While there is no consensus about whether insects are sentient, the possibility alone raises important questions. The AZA's guidelines state that it “is important that zoos and aquariums accredited by the [AZA] make certain that feeder animals are treated humanely and ethically while in their care” (AZA Nutrition Scientific Advisory Group 2017). Is it also important to treat *possibly* sentient animals humanely and ethically? If so, what does that involve? What ethical responsibilities, if any, do zoos and aquariums have concerning their feeder insects?

There has been very little scholarly discussion of these questions (essentially, just Keller 2017, which focuses on invertebrates' cognitive capacities and some possible welfare improvements; for some discussion of noninsect invertebrates, see Doerr and Stoskopf 2019 and Millar et al. 2023). This is not surprising, as scholars have largely ignored feeder animals generally (with a few notable exceptions: see, e.g., Cottle et al. 2010; Cooper and Williams 2014, Ings et al. 1997; Marshall et al. 2019; Di Marzio et al. 2023). So, this paper takes up two tasks.

First, it surveys the main welfare‐focused ethical questions associated with feeder animals generally and feeder insects in particular. (Accordingly, this paper brackets many other important but separate issues—e.g., the sustainability of sourcing particular feeder animals from the wild.) The aim here is to identify the main considerations that bear on a full assessment of the ethics of using feeder animals as a resource for future research.

Second, in the interest of making practical recommendations in the face of significant moral complexity and uncertainty, this paper adopts a standard institutional ethical framework—the 3Rs—and considers its implications for the use of feeder insects. The 3Rs framework recommends *replacing* animals with non‐animal models, *reducing* the number of animals used, and *refining* practices to minimize distress. Zoos and aquariums already use this ethical framework in some contexts and can readily extend it to others.

## 2 Ethical Questions about Feeder Animals

The ethics of rearing and using feeder animals touches on some of the foundational questions in animal ethics (Fischer 2021). However, these issues have yet to be laid out systematically in this context. So, we begin by identifying questions that apply to all feeder animals; then, we turn to insects specifically. The aim of this section is to demonstrate the complexity of the ethical issues and pave the way for future research on this topic.

### 2.1 General Ethical Questions

First, people often observe that it is “natural” for predators to consume prey (and often live prey). To what extent does this “naturalness” matter in our ethical deliberations? Do the circumstances of captivity change what counts as natural and unnatural? Does the intentional creation of animal life—as in a breeding program—change what counts as natural and unnatural? (For discussion, see Learmonth 2019.)

Second, feeding involves sacrificing the interests of relatively many animals for the sake of relatively few. Over the lifespan of a predator, that animal may consume thousands of other animals. (Given standard feeding recommendations for ball pythons, for instance, one python might eat 2000–3000 mice over their lifespan; Zayas 2024.) When, or to what degree, is this tradeoff warranted?

The answer to this second question depends on several others. The most important of them concern (a) the degree to which it is ethically acceptable to use some individuals to benefit others without their consent and (b) when it is ethically acceptable to bring new animals into existence. Issue (a) marks one of the fundamental divides in animal ethics, where some theories impose—and other theories do not impose—a consent‐based constraint on the use of animals that roughly mirrors the one that is generally accepted for humans (Fischer 2021). If there is a consent‐based constraint on the use of animals, then it is objectionable even to feed one animal to another. If there is not such a constraint, then it could work out that even large numbers of prey animals can be sacrificed for just one predator.

Issue (b) turns on one of the core questions in population ethics (Greaves 2017). According to one standard theory in population ethics, totalism, as long as animals have lives that are positive on balance (even if just barely), then there is nothing objectionable about bringing them into existence. On a rival theory, the critical level theory, it is objectionable to bring animals into existence unless the quality of their lives exceeds some higher threshold, the precise character of which is, perhaps not surprisingly, controversial. According to both views, it is objectionable to bring animals into existence if they have lives that are bad on balance. However, the views will disagree about the level of care necessary to make it acceptable to create additional animals, with a critical level theory having a higher standard than totalism.

To get the third and fourth questions on the table, we should consider the standard justifications for live feeding. First, people cite the nutritional needs of the predators, insofar as they will not take dead prey. Second, people cite live feeding as an enrichment for predators (Fens and Clauss 2024). The third question, therefore, is whether it is true of a given predator that the animal *requires* live prey to satisfy its nutritional needs, as many animals may be trained to accept dead prey, versus *preferring* live prey. Relatedly, we might be unsure as to what constitutes having taken reasonable steps to encourage predators to accept dead prey.

Fourth, if feeding on live prey is an enrichment, we might ask about the average *magnitude* and *replaceability* of the benefit that this enrichment provides. After all, not all enrichments are created equal: some are strongly preferred over others (as zoo professionals recognize; Mehrkam and Dorey 2015). Moreover, whatever the value of an enrichment, we ought to consider whether there are other ways of providing welfare‐enhancing stimulation that do not involve the distress of another animal. (That being said, we should recognize individual variation, where some individuals will benefit more and others less, which may also mean that some enrichments are more or less replaceable for particular individuals.)

Many of these questions about tradeoffs lead to a more foundational issue about comparing welfare impacts across species—one made more complicated by the different kinds of welfare impacts at issue. That is, given some estimates of the magnitudes of the welfare impacts on predators and prey, how do we put them on the same scale? For instance, there are likely to be various cognitive and affective differences between mice and snakes; do those differences matter when comparing benefits to snakes against harms to mice? In addition, how should we weigh a longer‐term but potentially more modest psychological benefit to a predator—namely, the alleviation of boredom—against the intense but relatively brief suffering of prey?

### 2.2 Feeder Insects: New Complexities

Feeder insects add some additional ethical questions to this list.

The first concerns the extent to which precautionary measures are warranted. In general, it is widely held that precautionary measures are warranted when implementing them can reduce the risk of causing serious harm at a relatively minor cost (where “cost” is understood broadly, to include the investment of time and energy). This idea has been developed in great detail in environmental contexts (Pyhälä et al. 2010) but has also been adapted for cases where sentience is uncertain (Birch 2024). Applied to insects, a precautionary approach recommends avoiding actions that are likely to cause insects severe pain if, in fact, they are sentient, at least when avoiding those actions is not particularly burdensome.

However, there remain many questions about the practical implications of this idea in a given situation. It is unclear how likely it is that insects are sentient, it is unclear what is likely to cause insects severe pain if they are sentient, and it is unclear how to balance the value of mitigating the risk of causing severe pain against the costs of precautionary measures.

Second, insects change the scales of the tradeoffs between predators and prey. Again, each python might eat 2000–3000 mice over their lifespan—and those animals are typically euthanized prior to feeding, avoiding concerns about additional suffering due to predation. Given standard feeding recommendations for bearded dragons, by contrast, each bearded dragon might eat 20,000–30,000 crickets over their lifespan and none of those crickets will be euthanized in advance. To what degree does this difference matter?

Third, some of the uncertainties mentioned in the previous section are deeper for insects. Insect welfare is a young field that has answered just a few questions about select species. So, we are a long way from understanding the welfare impacts of predation on many feeder species—much less estimating their magnitudes. Moreover, insect cognition is understudied, which means that there are large knowledge gaps about some of the capacities that might be relevant to comparing welfare impacts across species (though see Gibbons et al. 2022 and Fischer 2024 for reviews of many possibly relevant traits in some taxa).

## 3 A Starting Point: The 3Rs

Given the space available, it is not possible to explore all these questions in detail. But if we did, we'd quickly see that competing ethical frameworks give strikingly different answers. These debates are certainly worthwhile. However, in the interest of tethering this essay to a framework with some institutional purchase, let's consider these questions from the perspective of the 3Rs. The 3Rs, again, are the cornerstone for ethical animal research, recommending replacing animals with non‐animal models, reducing the number of animals used, and refining practices to minimize distress. But while designed for animal research, the 3Rs can be applied to any context where animals are kept or used for some purpose. When zoos are establishing or revising their protocols, the 3Rs can provide valuable guidance relative to their conservation and educational aims (Brando and Gjerris 2022).

It is essential to appreciate that the 3Rs are a relatively low bar, as they only require preventing unnecessary harm *given specific objectives*. To clarify this point, it is helpful to compare the 3Rs with harm–benefit analysis. Harm–benefit analyses only recommend policies where the benefits outweigh the harms. The 3Rs, by contrast, take an objective as given and only require minimizing harm relative to that objective. The 3Rs, therefore, are less demanding than harm–benefit analysis. It is possible to follow the 3Rs while pursuing research that does not produce a net benefit; however, it is not possible to follow the recommendations of a harm–benefit analysis while pursuing research that does not produce a net benefit.

To be clear, the claim here is *not* that keeping obligate carnivores does not produce a net benefit—whether in terms of welfare, conservation goals, educational goals, or anything else. Instead, the claim is about what does and does not need to be justified when we take the 3Rs as our ethical starting point. The 3Rs do not require us to justify the decision to keep predators in captivity, given that this means that other animals will be required as feed. The 3Rs only require us to justify how many feeder animals we use and how we treat them. This allows us to bracket some of the thornier questions mentioned above—for example, about naturalness and about sacrificing the many for the few.

Moreover, a *precautionary* approach to the 3Rs is more modest still. The 3Rs framework calls for minimizing unnecessary harm relative to our aims. Precautionary reasoning, by contrast, calls for taking steps to reduce risks that are proportionate both to the likelihood and severity of those negative outcomes. So, a precautionary approach to the 3Rs recommends taking steps to minimize risks that are proportionate both to the likelihood and severity of negative outcomes. That is, a precautionary approach to the 3Rs does not recommend minimizing risks per se; it calibrates the level of investment in risk reduction to the magnitude of the risk.

With these clarifications in mind, we can now consider what might be involved in applying precautionary reasoning when it comes to feeder insects.

## 4 Applying Precautionary Reasoning

To apply precautionary reasoning, we first need to consider what is appropriate when we are *not* (or at least *less*) uncertain about sentience. To that, let's consider the basics of treating any feeder animal humanely and ethically, as the AZA recommends. For the most part, the standards of care for feeder animals are identical to those for other captive animals. Their primary enclosures need to be suitable to their needs. Food and water should be provided regularly. Opportunities for social interaction should be provided for species that benefit from it. Animals should be monitored regularly, ensuring that behavior is normal and there are no signs of disease. There should be clear standards for euthanizing (vs. providing curative measures for) sick, injured, or otherwise irreparably distressed animals. Records should be maintained to ensure that animals' needs are never overlooked. And so on.

The best practices for using feeder animals are also fairly straightforward. If feeder animals can be euthanized prior to being fed to predators, then they ought to be—and in a way that minimizes distress. Sometimes, however, they cannot be. Of course, being hunted and consumed alive may well involve considerable distress and suffering. (While time to death is a coarse proxy for the amount of suffering, Cooper and Williams (2014) report that when reptiles eat mice, the mice can continue moving for up to 2 min after being caught.) So, steps should be taken to mitigate feeder animals' distress. For instance, caretakers can add a relatively small number of feeder animals to a predator's enclosure at a time, ensuring that those animals are caught and killed relatively quickly. Likewise, they might euthanize any feeder animals that are injured but not eaten.

Let's now return to insects, recognizing uncertainty about their sentience. How, or to what degree, does this uncertainty change any of the guidance above?

Plausibly, it does not change the husbandry recommendations for feeder insects at all. This is because the recommendations above would be independently advisable for maintaining healthy feeder insect populations. So, it is unclear that there are many costs, if any at all, to be balanced against the benefit of reducing the risk of causing severe pain.

Things are more complicated with respect to using feeder insects for their intended purpose. Here, the practical recommendations largely depend on two considerations:

1. The probability that insects are sentient, where a higher probability makes it easier to justify precautionary measures; and
2. The general strength of the case for live feeding, which includes a mix of considerations ranging from the needs of predators to how burdensome alternative feeding protocols would be for animal care professionals.

### 4.1 The Probability That Insects Are Sentient

It is, of course, enormously difficult to assign a specific probability to the hypothesis that any particular insect is sentient, with enormous additional complexities once we recognize the diversity of insect species and the variation across insect life stages. However, precision is not required for many practical purposes; therefore, we should not be overwhelmed by the challenge of achieving it. Instead, we should recognize that it is often enough to bound our estimate in a way that is relevant to making decisions.

Here are two questions we can ask to bound our estimate. First, is the probability negligible? Second, is the probability higher, lower, or on a par with the probability that the relevant predator is sentient?

Presumably, most readers of this paper will not think that the probability of insects being sentient is negligible. This suggests that some precautionary measures are justified. It is also plausible that, in most cases, readers of this paper will think that the probability that a particular insect is sentient is lower than the probability that the relevant predator is sentient. This suggests that, all else equal, justified precautionary measures will be less stringent than those measures that are justified for the relevant predator species. While these two points leave considerable room for disagreement about the specific precautionary measures that are justified, they are still action‐guiding, ruling out some possibilities that might otherwise have seemed reasonable: namely, no precautionary measures at all, on the one hand, and completely equal treatment, on the other.

### 4.2 The General Strength of the Case for Live Feeding

Several factors bear on the general strength of the case for live feeding. They include:

- Whether predators require live prey,
- The relative welfare benefits and costs of live feeding,
- The replaceability of live feeding as an enrichment, and
- The burdensomeness of alternative feeding protocols.

We review these considerations in turn.

#### 4.2.1 Whether Predators Require Live Prey

Live feeding is clearly advisable if predators require it to survive (recalling that the 3Rs do not challenge the objective of keeping those predators in the first place). However, we can not infer that a given adult predator requires live prey from the fact that the animal ignores dead prey. The assessment is more complex.

First, we need to distinguish between genuine requirements and strong preferences that might be overcome through training. Second, we should consider how developmental factors may influence what is possible in terms of training.

For many insectivorous species, the movement of prey serves as a feeding stimulus. This is particularly evident in amphibians and reptiles, where motion detection often triggers the feeding response. However, while there are no published surveys on the topic, there is ample anecdotal evidence that many predators can be conditioned to accept dead prey through various techniques, such as wiggling prey with forceps or gradually transitioning from live to freshly killed prey. This suggests that what appears to be a requirement may sometimes be modified through conditioning. Moreover, the timing of conditioning efforts may be crucial. For all we know, there are developmental windows when some predators can learn to accept dead prey even though they would reject it later in life. So, those working with younger animals may have opportunities that are worth exploring.

Still, these points alone do not show that the 3Rs recommend trying to train predators to accept dead insects. This is partly because we have not yet considered the burden on animal care professionals of training predators to accept dead insects (which, in all likelihood, is not uniform across species). It would be quite a lot of work for animal care professionals to (try to) train each predator to accept dead prey (see 4.2.4, below). In addition, we cannot yet draw any conclusions about what the 3Rs recommend because the burden of precautionary measures on *predators* requires some consideration. After all, in most cases involving insects, we are likely to be more confident that predators are sentient than prey. (This is not uniformly true, as we may be equally uncertain about some arthropod predators and their prey. However, the point likely applies to mammalian, avian, reptile, and amphibian insectivores.) So, predators and prey are not on equal footing here: there should be some priority for predators if, in fact, we are more confident that they will bear some welfare costs if live feed is not provided.

#### 4.2.2 The Relative Welfare Benefits and Costs of Live Feeding

The welfare cost is clear for predators that genuinely require live prey: they will starve to death. So, let's limit our focus to predators that will accept dead prey. Then, the challenge is to weigh the benefit of enrichment to predators against the costs to prey insects.

The benefits of enrichment, presumably, are largely the opportunity to engage in species‐typical behaviors and the alleviation of boredom. The costs to prey insects, if they are sentient, are the stress of being hunted and the pain associated with being consumed alive. However, welfare benefits and costs are rarely quantified in a way that readily allows us to compare them. A second problem is that, even if they were, we would face disagreements about what matters for welfare, creating uncertainty about how to compare different welfare benefits.

On the first point, welfare frameworks generally employ ordinal scales, where welfare states are ranked but not assigned (even roughly estimated) magnitudes. So, these frameworks do not let us say that the total welfare benefit of a given enrichment is a 2/10, the welfare cost of acute suffering is −8/−10, and thus the welfare cost of acute suffering exceeds the welfare benefit of a given enrichment (Fischer 2025).

On the second point, note that there is an important distinction between welfare assessment frameworks and theories of welfare. Welfare assessment frameworks, like the Five Domains framework, attempt to group welfare indicators into general categories to structure and improve welfare assessment. Theories of welfare are philosophical theories about the ultimate nature of welfare. The latter is our focus here. One common theory of welfare is pluralistic, where welfare has several dimensions, such as health, natural behavioral expression, and the experiential states of the animal (Broom 2022). A rival view is that welfare is about animals' experiences, where positive experiences contribute positively to the animal's welfare and negative experiences detract from the animal's welfare (Browning 2020). On the former view, it can be good for an animal to engage in natural behavior even if it does not contribute to the animal's health or positive experiences; each dimension of welfare is independent. On the latter view, the value of health and natural behavior is grounded in their impacts on the animals' experience. So, if natural behavior is valuable, it is because animals prefer it to the alternative.

It is difficult enough to know how to compare the experiences of animals (Fischer 2024). It is harder still if we need to weigh competing dimensions of welfare. So, there may not be anything uncontroversial to say about the relative welfare impacts of enrichment for predators and pain and/or distress for prey. However, that observation alone can be informative. If we are uncertain about how to compare these welfare benefits, then, for practical purposes, we should treat them as being on a par. But if they are on a par, then the 3Rs clearly side with prey. The 3Rs recommend both reducing the number of animals affected and refining procedures to avoid unnecessary harm. These goals would be in tension if predators' benefits from live feeding were enormous and the harms to prey were trivial. Then, favoring prey would remove a substantial enrichment, leaving predators much worse off for relatively little gain. However, if we should treat the welfare impacts symmetrically, then the call to reduce the number of live animals used is decisive.

As mentioned above, however, we should not treat the welfare impacts symmetrically, at least given that we are more confident that the relevant predators are sentient than that the relevant insects are sentient. Still, the number of individuals can be relevant. Even if we place greater weight on the interests of predators, the sheer number of insects affected may make precautionary measures appropriate.

#### 4.2.3 The Replaceability of Live Feeding as an Enrichment

Even if predators do not require live prey, they may prefer it. This raises the question of whether that welfare benefit—or a comparable one—can be secured via other means. Let's suppose that live feeding provides enrichment along several axes: it provides cognitive stimulation through hunting behavior, offers sensory enrichment through prey movement and scent, and enables the expression of species‐typical behaviors. At least some of these benefits are probably achievable in other ways. For example, cognitive stimulation could be provided through puzzle feeders or other foraging enrichment devices.

Granted, the effectiveness of such alternatives likely varies across species and individuals. For some predators, perhaps those with more elaborate hunting behaviors, the replacements might provide only partial substitutes for the enrichment value of live prey. For others, alternative enrichment strategies might prove equally satisfying while avoiding welfare costs to prey insects.

#### 4.2.4 Burdens on Animal Care Professionals

Animal care staff have limited time as it is. They are under significant pressure to provide high‐level care for many animals with unique needs. This complicates the case for possible refinements to standard operating procedures. It is implausible, for instance, that staff could go through individual training protocols with each insect‐consuming animal without additional support.

That being said, precautionary measures will vary considerably in how burdensome they are. For instance, some predators may be willing to accept dead prey with training. In any given case, this may only take a few minutes. However, while any particular action may not be very burdensome, a holistic change still could be. The process would need to be repeated many times for the many different species that consume insects. So, one realistic precautionary measure may simply be: feeding dead insects to those predators that will accept them without any training.

This suggests that, while individual animal care professionals have ethical reasons to look for ways of refining their practices, the details may largely be determined by practical constraints, making it difficult to issue general recommendations. However, at the organizational level, zoos and aquariums have ethical reasons to increase staffing levels to make better practices possible. Just as researchers ought to secure the staffing required to comply with the 3Rs, zoos and aquariums ought to secure the staffing required to comply with the 3Rs.

## 5 Conclusion

Given specific objectives, the 3Rs recommend replacing animals with non‐animal models, reducing the number of animals used, and refining practices to minimize distress. In the context of a discussion about feeder insects, the relevant “Rs” are reduction and refinement. Is it possible to refine animal husbandry practices so that feeder insects do not suffer any unnecessary distress? Is it possible to refine feeding procedures to reduce unnecessary harm?

The answer to the first question is likely affirmative, given that many best practices for animal welfare align closely with best practices for animal health. The answer to the second question is more complicated. What counts as a reasonable precautionary measure depends on the probability that insects are sentient and the general strength of the case for live feeding. The higher that probability, the stronger the case for precautionary measures. And where animals can readily be fed euthanized insects, there is a strong case for that being the best practice.

Crucially, it is important not to accept the status quo in terms of staffing—or anything else—when trying to provide a high standard of animal care. Zoos and aquariums aim to be leaders in captive animal welfare. Their mission gives them strong reasons to consider how best to implement precautionary measures based on the realistic possibility that their feeder insects are sentient. While many of the ethical issues here are controversial, the 3Rs are simple, consensus‐generating tools for navigating the key uncertainties. When applied carefully to the issue of live‐feeding insects to predators, they appear to support the same kinds of recommendations that are now standard for other feeder animals: namely, avoiding live feeding when possible and trying to mitigate distress when it is not possible.

## Ethics Statement

The research for this paper did not involve any human or animal subjects; therefore, no ethical approval was required.

## Data Availability

The author has nothing to report.
